# *rps3* as a Candidate Mitochondrial Gene for the Molecular Identification of Species from the *Colletotrichum acutatum* Species Complex

**DOI:** 10.3390/genes11050552

**Published:** 2020-05-14

**Authors:** Agnieszka Pszczółkowska, Piotr Androsiuk, Jan Paweł Jastrzębski, Łukasz Paukszto, Adam Okorski

**Affiliations:** 1 Department of Entomology, Phytopathology and Molecular Diagnostics, University of Warmia and Mazury in Olsztyn, ul. Prawocheńskiego 17, 10-720 Olsztyn, Poland; agnieszka.pszczolkowska@uwm.edu.pl (A.P.); adam.okorski@uwm.edu.pl (A.O.); 2 Department of Plant Physiology, Genetics and Biotechnology, University of Warmia and Mazury in Olsztyn, ul. Oczapowskiego 1A, 10-719 Olsztyn, Poland; bioinformatyka@gmail.com (J.P.J.); pauk24@gmail.com (Ł.P.)

**Keywords:** *Colletotrichum fioriniae*, *Colletotrichum lupini*, *Colletotrichum salicis*, *Colletotrichum tamarilloi*, comparative genomics, phylogenetic relationships, NGS, species identification

## Abstract

*Colletotrichum* species form one of the most economically significant groups of pathogenic fungi and lead to significant losses in the production of major crops—in particular, fruits, vegetables, ornamental plants, shrubs, and trees. Members of the genus *Colletotrichum* cause anthracnose disease in many plants. Due to their considerable variation, these fungi have been widely investigated in genetic studies as model organisms. Here, we report the complete mitochondrial genome sequences of four *Colletotrichum* species (*C. fioriniae, C. lupini, C. salicis,* and *C. tamarilloi*). The reported circular mitogenomes range from 30,020 (*C. fioriniae*) to 36,554 bp (*C. lupini*) in size and have identical sets of genes, including 15 protein-coding genes, two ribosomal RNA genes, and 29 tRNA genes. All four mitogenomes are characterized by a rather poor repetitive sequence content with only forward repeat representatives and a low number of microsatellites. The topology of the phylogenetic tree reflects the systematic positions of the studied species, with representatives of each *Colletotrichum* species complex gathered in one clade. A comparative analysis reveals consistency in the gene composition and order of *Colletotrichum* mitogenomes, although some highly divergent regions are also identified, like the *rps3* gene which appears as a source of potential diagnostic markers for all studied *Colletotrichum* species.

## 1. Introduction

Members of the genus *Colletotrichum* pose one of the greatest threats to the health of host plants around the world. According to previous research, *Colletotrichum* species are the eighth most economically significant group of pathogenic fungi in the world [1,2,3]. Nearly all cultivated plants are susceptible to infections caused by one or more species of *Colletotrichum* [1,4]. These fungi cause significant losses in the production of major crops—in particular, fruits, vegetables, ornamental plants, shrubs, and trees. Members of *Colletotrichum* cause anthracnose, a disease that leads to the formation of dark spots on the aboveground plant parts (leaves, shoots, and pods) and causes fruit and vegetable rot [1,4,5,6,7]. *Colletotrichum* species also cause losses in agricultural raw materials and crops stored after harvest, due to undetected latent infections that develop during storage [7,8].

There are many blind spots in the taxonomy of the pathogenic fungi of *Colletotrichum* due to differences in the applied evaluation criteria. The reported number of *Colletotrichum* species ranges from 29 to more than 700 [1,9,10], and 889 records have been entered into the Index Fungorum database to date [11] The genus *Colletotrichum* has been regarded as a model organism for hemibiotrophic pathogens, with a brief biotrophic phase followed by a necrotrophic phase of infection [1]. *Colletotrichum* species rely on various infection strategies, including biotrophic, necrotrophic, hemibiotrophic, and endophytic, to colonize the tissues of a host organism and derive nutrients [7,12]. Due to their considerable variations, these pathogens have also been widely investigated as model organisms in genetic studies. Multilocus phylogenetic studies of *Colletotrichum* support the presence of at least 10 large clades, including *C. acutatum*, *C. gloeosporioides,* and *C. boninense* species complexes comprising at least 28, 22, and 17 species, respectively [5]. *Colletotrichum* species identified within the major clades differ significantly in their ability to infect host plants. The *C. acutatum* complex includes polyphagous species such as *C. nymphaeae*, *C. simmondsii,* and *C. fioriniae,* as well as *C. salicis,* which has a preference for woody hosts [6], *C. lupini,* which infects lupines [13], and *C. tamarilloi,* which only targets potatoes [6]. Pszczółkowska et al. [14,15] isolated *C. fioriniae* from highbush blueberry and common beech. The cited authors also identified *C. lupini* in the seeds of various lupine species [16,17]. Okorski et al. [18] confirmed an infection caused by *C. salicis* in willow shoots based on morphological identification and an analysis of selected gene sequences. Baroncelli et al. [19] reported on the plasticity of fungal genomes and observed that changes in gene structures are largely influenced by the spectrum of host plants. The *C. acutatum* species complex is varied but is also closely related to the group of fungal plant pathogens. It is characterized by significant variations in major traits, including the range of host plants, host preferences, mode of reproduction, and infection strategies. In comparative analyses of fungal genomes, attempts have been made to link specific genes with the evolutionary patterns of fungal species [20]. The next point of concern is the precise identification of fungal species. Although, for many species, a multilocus DNA barcode system is available for which both mitochondrial and nucleic sequences have been exploited [21], there are still a number of taxa, including those from the genus *Colletotrichum*, for which there is a strong need to develop an efficient tool for phylogenetic inference and species identification [6].

The aim of this study was to report the complete mitochondrial (mt) genome sequences of four *Colletotrichum* species (*C. fioriniae, C. lupini, C. salicis,* and *C. tamarilloi)* and to compare them with other publicly available mt genomes of the *Colletotrichum* species to identify the characteristic features of the sequenced genomes and their possible applications in genetic diversity studies and species identification.

## 2. Materials and Methods

### 2.1. Fungal Isolate and DNA Extraction

The *Colletotrichum* isolates used in this study were derived from various plant species collected in Poland and from the CBS (Central Bureau of Fungal Cultures) Westerdijk Fungal Biodiversity Institute, Utrecht, the Netherlands. *C. lupini* CBS 119142 was derived from *Lupinus albus* in Elsenburg (South Africa, 1995), *C. fioriniae* isolate Cf.60.014.DDPP was derived from *Vaccinium corymbosum* leaves (Poland, 2014), *C. salicis* SP17/2016 was isolated from *S. vinimalis* × *S. schwerini* shoots (Poland), and *C. tamarilloi CBS 129814* (*Solanum betaceum* was taken from fruit anthracnose, Cundinamarca (Colombia, 2012). Fungal cultures were grown in 90 mm sterile Petri dishes with PDA (Potato Dextrose Agar) medium. For DNA extraction, the mycelium of the species studied was scraped off the plates. Genomic DNA was extracted from 10-day *Colletotrichum* cultures, which were grated (homogenized) using a pestle and mortar in liquid nitrogen and proceeded according to the protocol using a Maxwell® 16 FFS Nucleic Acid Extraction System, Customs X9431 (Promega GMBH, Madison, WI, USA).

### 2.2. DNA Sequencing, Assembly, and Annotation of the Mitogenomes

Genome libraries were prepared from the genomic DNA using a Nextera XT kit (Illumina Inc., San Diego, CA, USA). Prepared libraries were sequenced on the Illumina MiSeq Platform (Illumina Inc., San Diego, CA, USA) with a 150 bp paired-end read. The trimmed reads were mapped to the reference of the complete mitochondrial genome of *Colletotrichum acutatum* using Geneious Mapper (ver. 8.0.4) [22] with “Medium-Low Sensitivity” parameters. Reads aligned to the reference mtDNA genome were extracted and used for de novo assembly (K-mer—23–41, low coverage cut-off—5, minimum contig length—300) separately for each *Colletotrichum* species. De novo contigs were extended by mapping raw reads to the generated contigs, reassembling the contigs with mapped reads, and manually scaffolding the extended contigs (minimum sequence overlap of 50 bp and 97% overlap identity). This process was iterated five times. Finally, the reduced sequences were assembled in the circular mitochondrial genome for each of the four species. The mitochondrial genomes were annotated using MFannot [23] and PlasMapper [24] with manual adjustments.

### 2.3. Characteristic and Comparative Analysis of the Mitogenomes

The complete mitochondrial genomes of *Colletotrichum fioriniae*, *C. lupini, C. salicis,* and *C. tamarilloi* from the *C. acutatum* species complex were characterized in terms of their size and composition. Furthermore, the gene contents and orders of the four referred mitogenomes were compared to those of previously published mitochondrial genomes of other representatives of the genus *Colletotrichum*. For that purpose, the mitogenomes of seven *Colletotrichum* species were downloaded from GenBank: *C. acutatum*, four representatives of the *C. gloeosporioides* species complex (*C. aenigma, C. fructicola*, *C. gloeosporioides*, and *C. siamense*), *C. graminicola* (*C. graminicola* species complex), and *C. lindemuthianum* (*C. orbiculare* species complex) (Table 1). In order to analyze the interspecific variation among the five representatives of the *Colletotrichum acutatum* species complex, a comparison of their mitochondrial genomes was performed using the mVISTA program, with the application of the Shuffle-LAGAN mode [25]. *C. acutatum* was set as a reference. Moreover, to check whether the representatives of *Colletotrichum gloeosporioides* species complex, *C. graminicola* and *C. lindemuthianum*, share conserved regions with *C. acutatum*, their sequences were also included in the analysis. The comparison was performed on mitogenome sequences aligned by MAFFT v7.310 [26].

An analysis of the evolutionary rates of genes shared by the abovementioned 11 species of the genus *Colletotrichum* was also performed. The group of 15 genes was selected to estimate the ratio of non-synonymous (Ka) to synonymous (Ks) substitutions. These genes were extracted and aligned separately using MAFFT v7.310. The Ka and Ks for each of the shared genes were estimated in DnaSP [31]. Since the reported sequences of the *Colletotrichum* species belong to the *C. acutatum* species complex, all selected sequences from the analyzed mitochondrial genomes were compared with those from the mitogenome of *C. acutatum*.

### 2.4. Identification of Repetitive Elements

The REPuter program [32] was used to detect and assess genomic repeats, including forward, reverse, palindromic, and complementary sequences with a minimal length of 30 bp, a Hamming distance of 3, and 90% sequence identity. Mitochondrial simple sequence repeats (SSR) or microsatellites were identified in Phobos v.3.3.12 [33] with default settings for perfect SSRs with a motif size of one to six nucleotide units. Standard thresholds for the identification of mitochondrial SSRs were applied [34], i.e., a minimum of 12 repeat units for mononucleotide SSRs, six repeat units for dinucleotide SSRs, four repeat units for trinucleotide SSRs, and three repeat units for tetra-, penta-, and hexanucleotide SSRs.

### 2.5. Phylogenetic Analysis

Phylogenetic analyses were performed on sequences of 15 protein-coding genes shared by 46 fungi species belonging the Sordariomycetes class, including 11 *Colletotrichum* species and two species of *Penicillium* as an outgroup. The appropriate sequences were downloaded from the NCBI database (Table S1). The chosen sequences were aligned in MAFFT v7.310. Bayesian Inference (BI) and Maximum-Likelihood (ML) methods were used for genome-wide phylogenetic analyses in MrBayes v.3.2.6 [35,36] and PhyML 3.0 [37]. Before BI and ML analysis, the best fitting substitution model was searched for in Mega 7 [38], and the GTR + G + I model was selected. A BI partitioning analysis was carried out to develop a majority rule consensus tree with 1 × 10^7^ generations using the Markov Chain Monte Carlo (MCMC) method. The tree sampling frequency was 1000 generations. The first 2500 trees were discarded as burn-in, with a random starting tree. The ML analysis was performed in PhyML 3.0 with 1000 bootstrap replicates.

In order to infer the phylogeny of the *rps3* gene, an analogical approach was applied. However, in the case of the set of *rps3* sequences derived from 44 species representing Sordariomycetes and two *Penicillium* species, Mega 7 software marked out the GTR + G substitution model as the optimal model, and therefore, it was applied in the BI and ML analyses. A detailed comparison of *rps3* nucleotide sequences from 11 representatives of *Colletotrichum* was also performed. For this purpose, Geneious software was applied. As a consequence, polymorphic regions within the gene were identified and characterized.

## 3. Results

### 3.1. Genome Size and Organization

Four *Colletotrichum* species were sequenced to produce 1,557,270 (*C. lupini*), 1,769,902 (*C. tamarilloi*), 1,918,044 (*C. fioriniae*), and 4,983,140 (*C. salicis*) raw reads with lengths ranging from 150 to 300 bp. These were then mapped separately to the reference genome of *C. acutatum* (NC_027280). Obtained contigs were enlarged and assembled de novo to build the final complete circular mtDNA of each species. The six *Colletotrichum* mt genome sequences were then submitted to GenBank and acquired the following accession numbers: NC_030052 for *C. fiorinae*, NC_029213 for *C. lupini*, NC_035496 for *C salicis*, and NC_029706 for *C. tamarilloi.*

The complete mitochondrial genomes of *Colletotrichum fioriniae*, C. *lupini, C. salicis,* and *C. tamarilloi* appeared as circular molecules with sequence lengths ranging from 30,020 bp (*C. fioriniae*) to 36,554 bp (*C. lupini*) (Table 2). For *C. salicis* and *C. lupini,* the mitogenomes notably exceeded the average length of the mt genome observed within the *C. acutatum* species complex, which, for *C. acutatum, C. fioriniae, C. lupini, C. salicis* and *C. tamarilloi*, was 32,448 ± 2742 bp (SD). These species had mitogenomes closer to those found in *C. graminicola* and *C. lindemuthianum*. On the other hand, the average size of the mitogenome within the *C. gloeosporioides* species complex (estimated for *C. gloeosporioides, C. aenigma, C. fructicula,* and *C. siamense*) was almost two-fold bigger (1.72) than the average size of the mt genome within the *C. acutatum* species complex. The lengths of noncoding regions seemed to be the main contributors to the observed differences, as was shown by the very similar values of the total length of coding sequences (CDS) observed for the *C. gloeosporioides* species complex and the *C. acutatum* species complex.

The GC content within the *C. acutatum* species complex was found to be at a rather low level and spread within the range from 29.91% (*C. lupini*) to 30.5% (*C. tamarilloi*), similar to the values of that trait found for *C. graminicola* and *C. lindemuthianum*. Analogically to the mt genome size, the representatives of the *C. gloeosporioides* species complex were characterized by higher GC contents, which, in all cases, exceeded 34% (Table 2).

In order to analyze the overall sequence conservation, to detect the diverged regions in the mitochondrial genomes of representatives of the *C. acutatum* species complex (*C. acutatum, C. fioriniae, C. lupini, C. salicis, C. tamarilloi*), and to check whether the representatives of the *Colletotrichum gloeosporioides* species complex as well as *C. graminicola* and *C. lindemuthianum* share conserved regions with *C. acutatum*, the mVISTA tool was applied. The aligned sequences revealed high similarity for the mt genomes within the *C. acutatum* species complex and clear divergence between the *C. acutatum* species complex and other representatives of *Colletotrichum* (*C. gloeosporioides* species complex, *C. graminicola,* and *C. lindemuthianum*). Moreover, the coding regions appeared to be more conserved than their non-coding counterparts in all analyzed species (Figure S1).

**Figure 1:**
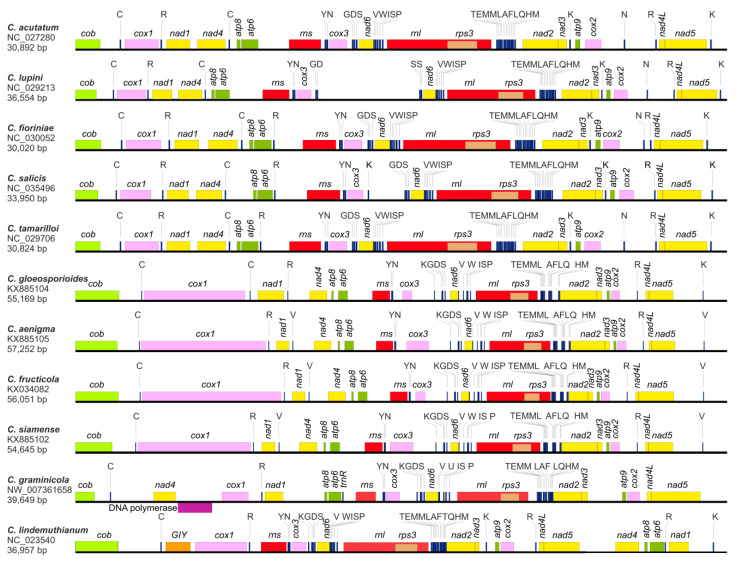
Linear maps of the mitochondrial genomes of 11 *Colletotrichuim* species. All genes, excluding DNA polymerase in the *C. graminicola* mt genome, are encoded on the same strand. Standard nomenclature was applied for protein and rRNA genes, whereas for tRNA genes, single-letter abbreviations were used. The presented maps include updated genome annotation data for *C. graminicola,* and *C. lindemuthianum* (striped bars for *rns* and *rnl*).

For all 11 analyzed mitogenome sequences, 14 protein-coding genes were found to be conserved among fungi, including seven subunits of the electron transport complex I (*nad1*, *nad2*, *nad3*, *nad4*, *nad4L*, *nad5*, and *nad6*), one subunit of complex III (*cob*), three subunits of complex IV (*cox1*, *cox2*, and *cox3*), and three subunits of the ATP-synthase complex (*atp6*, *atp8*, and *atp9*) (Figure 1). Moreover, the *rps3* gene, which encodes the 40S ribosomal protein S3, as well as genes for large and small ribosomal RNA (*rnl* and *rns*, respectively) were identified in all reported mt genomes. The sequence for the *rps3* gene was located within the intron of *rnl.* Reanalysis of mt genomes of *C. graminicola* (NW_007361658) and *C. lindemuthianum* (NC_023540) allowed us to identify previously unannotated *rnl* and *rns* in both species in the following locations: *rnl* (28,092–33,124) and *rns* (22,173–23,805) in *C. graminicola*, and *rnl* (15,745–20,963) in *C. lindemuthianum*. The order of the 15 abovementioned protein-coding genes and the two ribosomal RNA genes was highly conserved among the studied representatives of the *C. acutatum* species complex and *C. gloeosporioides* species complex. Some differences were observed in the gene order of *C. graminicola* and *C. lindemuthianum*, reflecting their taxonomical affiliations (Figure 1). Furthermore, for *C. graminicola* and *C. lindemuthianum*, annotations for two additional protein-coding sequences were found within their mitogenomes: DNA polymerase in *C. graminicola* [29] and GIY endonuclease in the case of *C. lindemuthianum* [30]. Reannotation of the *C. lindemuthianum* mt genome revealed the presence of a group IA intron containing hypothetical ORF within the *cob* gene; however, the presence of maturase in that location [30] was not confirmed.

All four *Colletotrichum* mitogenomes reported here (*C. fioriniae*, C. *lupini, C. salicis* and *C. tamarilloi*) included 29 tRNA genes (*trn*) that recognize codons for all amino acids; however, the composition differed slightly between species (Figure 1). For *C. fioriniae* and *C. tamarilloi*, there were 23 tRNA genes with a single copy and an additional four (*trnC*-GCA, *trnK*-TTT, *trnR*-ACG, and *trnN*-GTT) were doubled, whereas *trnM*-CAT had three copies. Within the mitogenome of *C. lupini,* there was a lack of one copy of *trnR*-ACG; however, an additional copy of *trnS*-GCT was found. An expansion in the number of copies of tRNA genes was also observed in *C. salicis* in which three copies of *trnK*-TTT were observed; however, this was accompanied by the deletion of one copy of *trnN*-GTT. The tRNA gene composition and order of *C. acutatum* was identical to that observed for *C. fioriniae* and *C. tamarilloi*, with one exception—the *trnR*-ACG gene was not present. For the remaining *Colletotrichum* species representatives, the number of tRNA sequences within the mitogenome ranged from 25 to 28, which was enough to provide a sufficient number of *trn* sequences that recognize codons for all amino acids (Figure 1). However, the exception was *C. graminicola,* where the *trn* for tryptophan was replaced by the *trn* for selenocysteine [29].

All mitochondrial genes of *C. fioriniae*, C. *lupini, C. salicis,* and *C. tamarilloi* were transcribed from the same DNA strand. The same analogical situation has been described for other sequenced mt genomes of *Colletotrichum* spp., with the exception of *C. graminicola,* where the sequence for DNA polymerase was encoded on a complementary strand. A detailed analysis of the mitogenomes showed that, in all studied species, there was only one example of sequence overlap: a 10 nt overlap in the *trnV*-TAC by the *nad6* sequence.

The majority of protein-coding genes start with the typical AUG codon and terminate with either UAA or UAG (*cox1*, *cox3* and/or *nad6*). However, in the case of *cox3* for *C. gloeosporioides*, *C. aenigma*, *C. fructicola,* and *C. siamense*, as well as in the *atp6* sequence in *C. graminicola,* alternative start codons, UUG and AUU, were found, respectively (Table 2). The codon usage frequencies were generally similar among all eleven *Colletotrichum* mitogenomes (Table S2). The most frequently used codons were UUA (L, 11.29–11.87%), AUA (I, 6.06–6.93%), and UUU (F, 5.1–5.81%). There were also codons (AUU, UUG, CGC, CGG, UGG, CUC, and AGG) found only in a portion of the studied mitogenomes, among which AUU deserves special attention, since it was found within only one species (*C. graminicola*). Furthermore, three codons CUC, UUG, and CGC have a potentially diagnostic character and can be used to distinguish the members of the *C. acutatum* species complex from those of the *C. gleosporioides* species complex, since they were found in only one group, while they were completely absent in the other.

Only one intron was found in the *C. fioriniae, C. lupini,* and *C. tamarilloi* mitochondrial genes, each time within the *rnl* sequence. An analysis of the mitogenome of *C. salicis* revealed the presence of two introns: one within *rnl* and one within the *rns* sequence. The remaining representatives of *Colletotrichum* may have up to eight or even nine introns within their mitogenomic genes [28].

**Figure 2:**
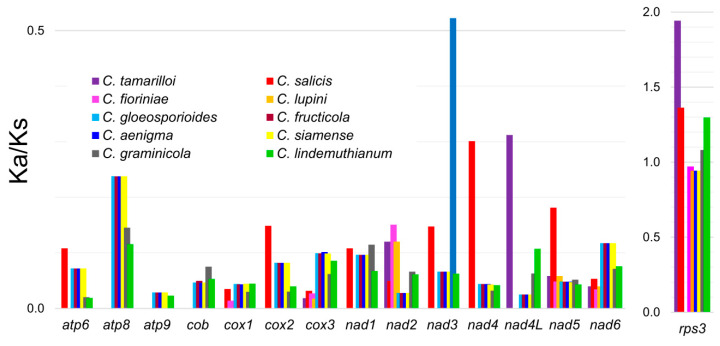
The distribution of non-synonymous (Ka) to synonymous (Ks) substitutions ratio among 15 protein-coding mitochondrial genes of eleven *Colletotrichum* species, using *C. acutatum* as reference genome. For *rps3*, due to its highest variation, different Ka/Ks ratio scale was applied.

To determine the nature of the evolutionary selection pressure in *Colletotrichum*, we estimated the evolutionary rates of 15 mitochondrial genes that are involved in electron transport complexes I, III, and IV, as well as the ATP synthase complex and *rps3* gene, which encodes 40S ribosomal protein S3. In this analysis, we included all 11 *Colletotrichum* species for which the complete mitochondrial genomes are currently available, including the mitogenomes reported here for *C. fioriniae, C. lupini, C. salicis,* and *C. tamarilloi*. Genes with non-applicable (NA) Ka/Ks ratios were changed to zero. The Ka/Ks ratio for most genes was less than one, with the exception of *rps3*. In the case of that gene, the Ka/Ks ratio was the highest for *C. tamarilloi* and *C. salicis*, for which it reached values of 1.942 and 1.363, respectively (Table S3a, Figure 2). A Ka/Ks ratio higher than one for *rps3* was also noted for *C. lindemuthianum* (1.298) and *C. graminicola* (1.081). In the case of the other genes, the Ka/Ks ratio in all analyzed species did not exceed the value of 0.523, which was noted for *nad3* in *C. graminicola*. A comparative analysis of genes revealed that the substitution rate varied widely, with Ka and Ks values ranging from zero to 0.218 and from zero to 1.036, respectively. The highest synonymous substitution rate (average Ks = 0.591) was observed for the *cox3* gene, whereas the lowest average Ks (0.108) was noted for *nad4L*. The highest average non-synonymous (Ka) substitution rate was observed for *rps3* (average Ka = 0.129), whereas the lowest was noted for *atp9* (0.005).

Based on Ka/Ks values, 14 genes indicative of purifying selection (Ka/Ks < 1) were identified in the analyzed mt genomes. A Ka/Ks ratio higher than 1.0 was only found for one gene (*rps3*), which is indicative of positive selection. In order to test whether traces of positive selection could be observed for the gene in all analyzed mitogenomes, pairwise comparisons of the Ka/Ks ratio for *rps3* were performed between all species combinations (Table S3b). In 15 out of 55 combinations, the Ka/Ks ratio was higher than 1.0, suggesting their adaptation to environmental conditions. Furthermore, in 34 other cases, the Ka/Ks ratio was slightly below one (>0,83), which may indicate at least some role of positive selection for *rps3* in the acceleration of the substitution rate.

### 3.2. Repeated Elements

A total of nine repeated sequences with lengths ranging from 31 to 205 bp and sequence identities greater than 90% (Table S4) were identified in *C. salicis*. In all cases, only forward repeats were scored. Most of the repeated sequences (6) were dispersed in the intergenic regions (IGS), and only three were localized within genes. For the other representatives of the *C. acutatum* species complex reported here (i.e., *C. tamarilloi*, *C. lupini,* and *C. fioriniae*), five repeated sequences were found in each mitochondrial genome. Their lengths ranged from 31 to 101 bp (*C. tamarilloi* and *C. lupini*) or from 41 to 151 bp (*C. fioriniae*). Analogically to *C. salicis,* only forward repeats were identified; however, this time, most of them (three to five) were localized within genes. The analysis of the previously reported *C. acutatum* mt genome [27] revealed the same number (five) of genomic repeats of a similar size, ranging from 31 to 112 bp, located predominantly (three) within the coding sequences. All of them were identified as forward repeats (Table S4).

An analysis of repeat sequences from the mt genomes of other representatives of *Colletotrichum* was also performed (Table S4). In the mitochondrial genome of *C. graminicola,* a total of 12 repeated sequences with lengths ranging from 30 to 75 bp were found. They included nine forward repeats and three palindromic repeats, all of them located within intergenic regions. The mitochondrial genome of *Colletotrichum lindemuthianum* included 21 repeat sequences with lengths ranging from 30 to 87 bp. All of them were identified as forward repeats located almost exclusively within the intergenic regions and introns (18). In the case of *C. gloeosporioides,* a total of 68 repeated sequences were found with lengths ranging from 30 to 107 bp. They included 40 forward repeats and 28 palindromic repeats located almost exclusively within intergenic regions and introns (65). Only three were identified within genes (*trnV*-TAC and *nad6*). An analysis of the mitogenome of *C. fructicola* allowed us to identify 54 repeated sequences with lengths ranging from 30 to 90 bp. They included 37 forward repeats and 17 palindromic repeats. Most of the repeated sequences were dispersed in the intergenic regions and introns (44), whereas 10 were identified within genes (*trnV*-TAC and large subunit of ribosomal RNA). For *C. siamense,* a total of 73 repeated sequences were found with lengths ranging from 30 to 83 bp. They included 46 forward repeats and 27 palindromic repeats located predominantly within the intergenic regions and introns (64), whereas nine were identified within genes (*trnV*-TAC and a large subunit of ribosomal RNA). The mitochondrial genome of *C. aenigma* included the highest number (124) of repeated sequences with lengths ranging from 30 to 100 bp. This included 66 forward repeats, 46 palindromic repeats, and 12 reverse repeats distributed mainly in intergenic regions and introns (107); only 17 were found within genes (*trnV*-TAC, *rps,* and a large subunit of ribosomal RNA).

The distribution and types of microsatellites were also studied in *Colletotrichum* mitogenomes (Table S5a–S5k). For all studied representatives of the *C. acutatum* species complex, the number of identified mitochondrial SSRs was rather low and ranged from 10 (*C. fioriniae*) to 18 (*C. lupini*). The di- and trinucleotide repeats appeared as the most abundant among the analyzed species, whereas penta- and hexanucleotide repeats were represented by solitary elements. A single mononucleotide repeat was only identified in the mt genome of *C. acutatum*. Regarding tetranucleotide repeats, five such elements were found in *C. lupini* and one in *C. tamarilloi* and *C. salicis*, while they were completely absent in *C. acutatum* and *C. fioriniae*. In comparison with those from the *C. acutatum* species complex, the representatives of the *C. gloeosporioides* species complex were characterized by a much higher number of SSRs found within their mitochondrial genomes—the number of these repetitive elements ranged from 31 (*C. siamense*) to 41 (*C. aenigma*). All identified SSR types occurred in the analyzed mitogenomes in various numbers and proportions, with some exceptions—the pentanucleotide repeats were not found in any of them; furthermore, hexanucleotide repeats were not found in the *C. fructicola* mitogenome. The remaining two *Colletotrichum* species, *C. lindemuthianum* and *C. graminicola*, were characterized by intermediate numbers of identified mitochondrial SSRs—25 and 27, respectively. For *C. graminicola,* all six SSR types were distinguished (mono-, di-, tri, tetra-, penta- and hexanucleotide); however, when *C. lindemuthianum* was considered, no hexanucleotide repeats were observed. Tri- and tetranucleotide repeats were the most abundant in these two *Colletotrichum* species.

The SSRs can be distributed across three different genomic regions: exons, introns, and intergenic spacers (IGS). For *C. lupini* and *C. fioriniae,* the SSRs were equally (50%/50%) distributed between exons and IGS (Table S5a–k). In other representatives of the *C. acutatum* species complex, the location of SSRs within exons predominated: 54.5% in *C. salicis*, 64.3% in *C. tamarilloi,* and 72.7% in *C. acutatum*. When the *C. gloeosporioides* species complex was considered, SSRs were found to be located predominantly in IGS (from 50% in *C. gloeosporioides* to 57.9% in *C. fructicola*) and were almost equally distributed within introns (range from 22.6% in *C. siamense* to 29.3% in *C. aenigma*) and exons (range from 18.4% in *C. fructicola* to 26.5% in *C. gloeosporioides*). The remaining two *Colletotrichum* species, *C. graminicola* and *C. lindemuthianum*, were characterized by having the highest number of SSRs located within IGS (59.3% in the former and 80% in the latter), and there was a complete lack of these repetitive elements within introns. A more detailed analysis of the SSRs in exons revealed that they could be found within the coding sequences of eleven genes (*nad1*–*nad6*, *cox1*, *cob*, *atp6*, *atp8* and large subunit ribosomal RNA), from which *cox1*, *nad2,* and *nad4* were the most common locations for identified microsatellites. On the other hand, *nad5* and *nad6* only appeared as SSR locations for *C. graminicola*. The joint analysis of all 11 *Colletotrichum* species revealed that among the identified mononucleotide repeats, the C/G motif was the most common (70.6%). For dinucleotide SSRs, the AT/TA motif was found to have the highest frequency (88.6%), whereas for trinucleotide repeats, two motifs, AAT/TTA (67.7%) and AAG/TTC (24.6%), were the most common. AGCC/TCGG (28.1%) and AAAT/TTTA (20.3%) were the most frequent tetranucleotide SSRs in all species. In pentanucleotide SSRs, three tandem repeat motifs were observed—AATAT/TTATA (77.8%), AAGCT/TTCGA (11.1%), and AAGAT/TTCTA (11.1%)—whereas in the case of hexanucleotide repeats, seven such elements were dominated by the AATGGG/TTACCC motif (36.4%).

### 3.3. Phylogenomic Analysis

**Figure 3:**
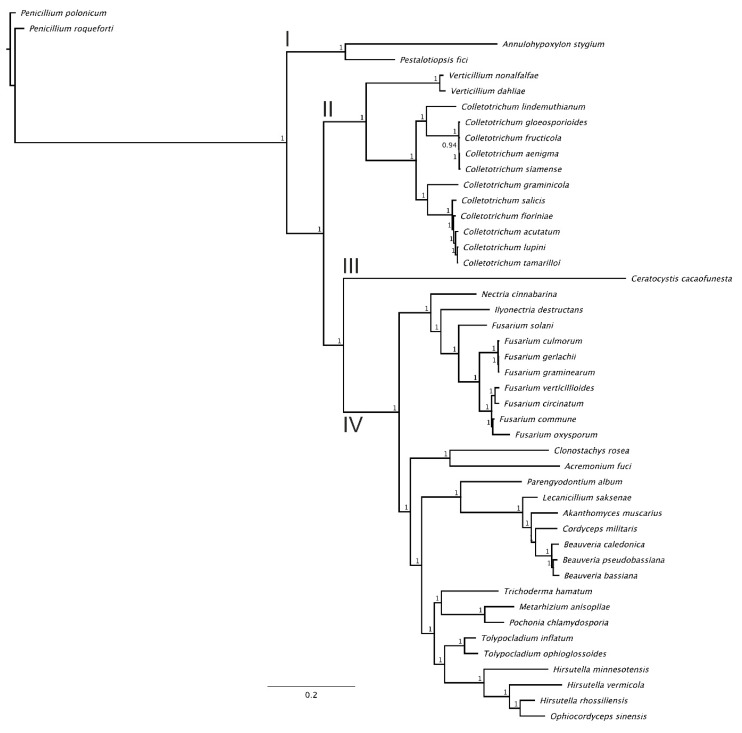
Phylogenetic tree based on sequences of 15 shared protein-coding genes from 11 *Colletotrichum* species, 33 other Sordariomycetes representatives, and two *Penicillium* sp., developed using Bayesian posterior probabilities (PP). Bayesian PP are given at each node.

The nucleotide sequences of 15 protein-coding genes were used for phylogenetic tree reconstruction. The ML and BI trees revealed consistent phylogenetic relationships. The BI tree (Figure 3) was characterized by very high nodal support values (only one node had a Bayesian posterior probability value below 1.0). Phylogenetic tree topology revealed systematic relationships between the analyzed fungi and divided them into four (I–IV) main clades. All analyzed *Colletotrichum* species were clustered into one clade (Clade II), and their topologies reflected their systematic positions: representatives of the *C. acutatum* species complex and *C. gloeosporioides* species complex formed two separate clusters, whereas *C. graminicola* and *C. lindemuthianum* formed solitary branches (the former appeared more similar to the *C. acutatum* species complex, whereas the latter was more similar to the *C. gloeosporioides* species complex). Two *Verticillum* species (*V. nonalfalfae* and *V. dahlia*) appeared as the most similar to the analyzed *Colletotrichum* species and, therefore, were found in the same clade. Clade III consisted of only one species: *Ceratocystis cacaofunesta*. Clade I joined *Annulohypoxylon stygium* and *Pestalotiopsis fici,* representing Sordariomycetes. The most heterogenous clade, Clade IV, contained 28 species, representing various genera from the Sordariomycetes class. Finally, two *Penicillium* species (*P. roqueforti* and *P. polonicum*), used here as the outgroup, occupied the most distinct positions on the dendrogram.

### 3.4. Molecular Evolution of rps3 in Colletotrichum

The analyzed *rps3* sequences varied in size among analyzed the *Colletotrichum* species, ranging from 1581 bp in *C. siamense* and *C. aenigma* to 1245 bp in *C. lindemuthianum.* However, for the *C. acutatum* species complex, the *rps3* length was very stable and fell within the range of 1416–1422 bp. A larger discrepancy was observed among representatives of the *C. gleosporioides* complex, where the length of *rps3* ranged from 1581 to 1329 bp (*C. fructicola*). When the other 35 fungi species were also considered (33 representatives of Sordariomycetes and two *Penicillium* species, *P. polonicum* and *P. roqueforti*, as an outgroup), the *rps3* length ranged from 1635 bp in *Hirsutella rhossiliensis* to 804 bp in *Penicillium polonicum.* Multiple alignment of all 46 *rps3* nucleotide sequences revealed the highest similarity of genes within the group of *Colletotrichum* species (on average, 78.55% identity), whereas the most divergent character (31.1% identity) was observed for the *rps3* sequence from the *Penicillium polonicum* mitogenome (Table S6). The average level of similarity between all analyzed sequences was 58.8%.

The nucleotide sequences of the *rps3* gene from the collection of all 46 fungi species were used for phylogenetic tree reconstruction (Figure S2). The ML and BI trees revealed consistent phylogenetic relationships. High consistency was observed for the topology of phylogenetic trees obtained on the basis of concatenated data for 15 protein-coding genes and sequences of the solitary *rps3* gene. In both cases, the positions of species reflected their systematic positions, with representatives of each genus generally gathered in the same cluster. This was also observed for the *Colletotrichum* genus, for which *rps3* appeared as a source of potentially diagnostic characteristics which could be applied in both evolutionary studies and for the development of markers to enable accurate species recognition.

A detailed comparison of the *rps3* sequence from 11 *Colletotrichum* species allowed us to identify a 223-bp-long polymorphic region suitable for the development of potentially diagnostic markers. This enabled us to distinguish all 11 studied *Colletotrichum* species. The abovementioned region was located between positions 529 and 751 of the *rps3* nucleotide multiple sequence alignment. It contained 140 polymorphic sites, resulting from either nucleotide substitution or insertion/deletion events (Figure S3). The characteristic distribution of indel polymorphism at positions 559–576 and 622–636 enabled the representatives of the *C. acutatum* complex and *C. salicis* to be distinguished from *C. lindemuthianum* and the *C. gloeosporioides* species complex. Moreover, unique indel polymorphisms were observed for *C. salicis* (546–551), *C. gloeosporioides* (583–609), *C. fructicola* (576–617), and *C. gramionicola* (619–645), which enabled them to be distinguished from each other and from the other representatives of *Colletotrichum*. For the other representatives of *Colletotrichum,* multiple alignments of their *rps3* nucleotide sequence revealed a number of polymorphic sites in the form of nucleotide substitutions that were characteristic of certain species (Figure S3).

## 4. Discussion

*Colletotrichum fioriniae*, C. *lupini, C. salicis,* and *C. tamarilloi* represent fungi with rather small mitochondrial genomes that do not exceed 37 kb. Although the complete mt genomes of these four species are, on average, almost three times larger than the smallest sequenced so far—the fungal mt genome of *Rozella allomycis*, Cryptomycota (~12 kb)—they are far smaller than the biggest fungal mt genome of *Rhizoctonia solani*, Basidiomycota (~235 kb) [39]. However, the size of the mitochondrial genome varies within the *Colletotrichum* genus and even reaches a length of 57,252 bp for *C. aenigma* or 58,666 bp for *C. siamense*, which are both representatives of the *C. gloeosporioides* species complex.

A number of studies have reported that the size variation of mitochondrial genomes may be caused by the length and organization of intergenic regions as well as by the presence of introns [40,41] which may vary in terms of their number and size. In some cases, the total length of intronic and intergenic regions may contribute up to 80% of the mt DNA sequence (*Phlebia radiata*; [42]). Intron content variation has been also reported as a source of variation in the size of mitogenomes, even among closely related species [43,44]. As was shown by our studies, compact mt genomes of representatives of the *C. acutatum species complex* include one (*C. acutatum*, *C. tamarilloi, C. fioriniae,* and C. *lupini*) or two (*C. salicis*) introns. However, for the *C. gloeosporioides* species complex, the number of introns was found to range from seven to nine, with the highest values found for *C. aenigma* and *C. fructicola*. Three of these introns were described as unique for all *C. gloeosporioides* sensu lato mitogenomes and thus potential targets for PCR-based detection [28].

The intergenic regions, as well as introns of the *Colletotrichum* mitogenomes, are filled with repetitive sequences, especially observed in the *C. gloeosporioides* species complex. For example, for the mitochondrial genome of *C. aenigma*, in which the highest numbers of palindromic, direct, and reverse repeats were found (124), 62.9% of them were found in intergenic spacers, whereas 31.5% were found in introns. On the contrary, the mitogenomes of the representatives of the *Colletotrichum acutatum* species complex are very poor in terms of their repetitive element content. The analysis of the mitochondrial genomes of *C. acutatum*, *C. fioriniae*, C. *lupini, C. salicis,* and *C. tamarilloi* revealed low numbers (five to nine) of only one type of repetitive element (forward repeats), and these were distributed predominantly (60–80%) in coding regions, with the exception of *C. salicis* for which repetitive elements within exons constituted 33.3% of their total number. The remaining two *Colletotrichum* species (*C. graminicola* and *C. lindemuthianum*) were characterized by an intermediate number of repetitive sequences (12 and 21, respectively); moreover, their locations were predominantly identified within intergenic regions (100% and 81%, respectively). An analogous situation was observed for all studied *Colletotrichum* species when the number and distribution of tandem repeats were considered.

Despite the identification of some differences in the size of the studied *Colletotrichum* mitogenomes, a high degree of consistency in their gene composition and order is observed. The *Colletotrichum* fungi generally contain a set of 17 genes in their mt genomes, including *atp6*, *atp8*, *nad4*, *nad1*, *cox1*, *cob*, *nad5*, *nad4L*, *cox2*, *atp9*, *nad3*, *nad2*, *rnl*, *rps3* (located within the intron of *rnl*), *nad6*, *cox3,* and *rns*. The abovementioned set of genes, together with sequences for 23 different tRNAs identified in the analyzed representatives of *Colletotrichum* (some of the *trn* genes were identified in more than one copy for certain species), fits the observation by Bullerwell and Lang perfectly [45], who stated that typical fungal mitochondrial genome usually encodes 30–40 genes. The highly conservative distribution of mitogenomic tRNA genes, which appeared to be organized in clusters across all analyzed *Colletotrichum* species, confirmed that the changes in *trn* locations are relatively rare events, and thus, an analysis of their distribution can be used to study fungal evolution or phylogenetic signals, including their contribution to gene order variation in fungal mitogenomes [46].

Nevertheless, some exceptions were found in the mt genomes of *C. graminicola* and *C. lindemuthianum*. For the mt genome of *C. lindemuthianum*, one additional gene was identified (GIY endonuclease) [30], whereas, in *C. graminicola*, a sequence of DNA polymerase was annotated [29]. For *C. lindemuthianum*, the annotated GIY endonuclease appeared as a free-standing ORF with a length of 1011 bp, placed between the sequences of *cox1* and *cob*. Contrary to the abovementioned unique element of the *C. lindemuthianum* mitochondrial genome, an additional sequence was annotated on the complementary strand of the *C. graminicola* mitogenome between the loci of *cox1* and *nad4* coded on the positive strand. This 2079-bp-long sequence, which encodes a protein with 692 amino acids, was identified as a member of the DNA polymerase type B protein family [29]. However, since all of the abovementioned additional genes annotated for *C. graminicola* and *C. lindemuthianum* were predicted solely based on the in silico analysis, we do not actually know whether they are active or, rather, if they are non-functional pseudogenes. Nevertheless, the unique characteristics of GIY endonuclease from *C. lindemuthianum* as well as DNA polymerase from *C. graminicola* makes them potential targets for diagnostic purposes as well as evolutionary studies, as has been proven previously for other systematic groups [47,48].

One of the main elements of evolution studies is the analysis of the substitution patterns within particular sequences [49]. Generally, synonymous substitutions dominate over non-synonymous changes within the coding sequences [50], and analogical patterns were identified in the mitogenomic genes of the 11 studied *Colletotrichum* species. The highest average synonymous substitution rate was observed for the *cox3* sequence. This unique characteristic of *cox3* was conditioned by the high Ks values estimated for all four representatives of the *Colletotrichum gloeosporioides* species complex, as well as for *C. graminicola* and *C. lindemuthianum*. In all of the abovementioned species, the Ks value was higher than 0.846, with *C. graminicola* having the highest value of 1.036. The opposite situation was observed for the *Colletotrichum acutatum* species complex, where the Ks values for *cox3* did not exceed 0.209. High polymorphism within the *cox3* sequence in mitogenomes of representatives of the *C. gloeosporioides* species complex was also previously reported by Liang et al. [28], who identified a short polymorphic region within the *cox3* sequence that showed strong lineage-specific divergence. The abovementioned 142 bp long region of *cox3* included 42.2% of all polymorphic sites present in the protein-coding regions of the analyzed mitogenomes and 64.3% of all nonsynonymous sites [28].

In our study, *rps3* appeared as the most variable nucleotide sequence with the highest number of non-synonymous substitutions. This was accompanied by high Ka/Ks values for almost all *rps3* sequences in *Colletotrichum* species in relation to *C. acutatum*, which was used as a reference. Four species (*C. tamarilloi, C. salicis, C. graminicola,* and *C. lindemuthianum*) had Ka/Ks values greater than one, suggesting that positive selection acts on *rps3* in *Colletotrichum* fungi. In another four *Colletotrichum* species (*C. aenigma, C. fructicola, C. siamense,* and *C. gloeosporioides*), the Ka/Ks value for the *rps3* sequence was within the range of 0.943–0.971. Although these values were <1, they were very close to the threshold value, which seemed to be a confirmation of that tendency. Our observations are concordant with the results of Lin et al. [51], who revealed a similar mechanism (positive selection) for *rps3* gene evolution for 12 representatives of Hypocreales fungi. When the remaining 14 protein-coding genes of 11 *Colletotrichum* mitogenomes analyzed in this study were considered, the Ka/Ks ratio did not exceed the value of 0.238, indicating that these sequences were under functional constraints, i.e., natural selection minimizes the number of amino acid changes to maintain the conservative characteristics of protein structure and function.

The abovementioned *rps3* gene, firstly described in yeasts as *var1*, is an optional element of many fungal mitogenomes [52,53]. *rps3* is the only ribosomal protein encoded in the mt genome that may be found either in the intron of the *rnl* gene or as a free-standing gene [54]. Furthermore, cytoplasmic (nuclear genome-encoded) versions of the rps3 protein have been reported [55,56]. In our study, *rps3* was found exclusively as an intronic ORF within the *rnl* gene. The *rps3* gene has become one of the key elements in fungal evolution studies, since its unique features have helped to reveal the mechanisms of the evolution or extinction of fungal mitochondria, including the mechanisms of fungal gene mobility within an organelle’s genome and/or the nucleus [57]. The data obtained for a number of fungal lineages also confirmed the importance of the *rps3* gene identified in phylogenetic studies of this group of organisms [54]. Generally, it is preferred that phylogenetic studies are performed based on data for several genes originating from nuclear or organellar genomes, or both [58,59,60]. However, as proved by Korovesi et al. [57] for 246 fungi species, the phylogenetic tree based on the sequences of only one gene (*rps3*) showed similar relations between studied lineages as the tree constructed based on concatenated data for 14 genes. Moreover, in our study, the phylogenetic tree for all 46 studied fungi species based on a solitary *rps3* sequence revealed a very consistent topology with the tree based on 15 mitochondrial protein-coding sequences. In both cases, the representatives of the *Colletotrichum acutatum* complex as well as the *Colletotrichum gloeosporioides* species complex formed two closely related but clearly separated clusters. *C. lindemuthianum* and *C. graminicola* formed solitary branches which revealed close relationship with the *Colletotrichum gloeosporioides* species complex and *Colletotrichum acutatum* species complex, respectively. The positions of other species reflected their systematic positions, with representatives of each genus generally gathered in the same clusters. The phylogenies based on data for a single gene usually represent the evolution of the respective gene only. However, some genes like *rps3* seem to provide a significant source of information on the evolution of the species carrying them. In our data, this can be observed for *Colletotrichum* or for species representing the same order, as revealed by Kang et al. [61] for Hypocreales.

Besides mitochondrial genes, a number of nucleic loci have been used for the determination of phylogenetic diversity and species identification within the *Colletotrichum* genus. The first phylogenetic studies of *Colletotrichum* were based on polymorphism within the internal transcribed spacer (ITS) region [62], which was considered as a standard barcode for the fungi. However, due to the insufficient level of polymorphism, the use of the ITS region to reveal species diversity within *Colletotrichum* has shown limited efficiency [63,64]. As a result, a number of other nuclear loci have been proposed and successfully used for multilocus phylogenetic inference and species identification within various *Colletotrichum* species complexes [64,65,66]. Very recently, a comprehensive evaluation of 11 of the most frequently used nuclear loci revealed that histone 3 (HIS3), glyceraldehyde-3-phosphate dehydrogenase (GAPDH), and β-tubulin (TUB2) are the best markers for *Colletotrichum* systematics and taxonomy [67]. However, for closely related fungi species for which the standard multilocus barcode is not sufficient, the utilization of the complete mitochondrial genome sequence as a super-barcode may have to be applied, in a similar manner to the approach successfully applied in plant genomics, where complete chloroplast genomes were recently proposed as a tool for taxonomic studies [68]. Nevertheless, the results of the current study for the phylogenetic relationships between *Colletotrichum* species based on concatenated data for 15 mitochondrial protein-coding sequences and the solitary mitochondrial *rps3* gene are in accordance with these revealed by nuclear loci. For example, only a slight difference in tree topology was observed when our phylogenetic trees were compared with those obtained for the *C. acutatum* species complex on the base of nucleic multilocus data which included ITS, GAPDH, CHS-1 (chitin synthase 1)>, HIS3, ACT (actin), and TUB2 [6]. This constitutes an additional argument for the potentially unique value of the *rps3* gene in the comparative genomics of *Colletotrichum*.

Here, we would also like to propose another novel approach for the utility of the *rps3* sequence in fungi genomics. The high variability identified within the *rps3* sequence among the studied *Colletotrichum* species encouraged us to perform a more detailed analysis of the studied sequence. As a result, we identified a polymorphic region of *rps3* that is suitable for the development of potential diagnostic markers that enable all 11 studied *Colletotrichum* species to be distinguished. This is an important issue since, despite strong efforts, *Colletotrichum* species are still identified based on the simultaneous analysis of several polymorphic loci [69,70,71]. The efficiency of the identification of fungi from that taxonomic group could be substantially improved by enhancing the available and commonly used set of mitochondrial and nucleic loci by the *rps3* sequence. Nevertheless, to confirm the effectiveness of the *rps3*-based diagnostic markers, further studies and tests on a wider number of samples for each *Colletotrichum* lineage are required.

## 5. Conclusions

More than ten genomes of the genus *Colletotrichum* have been sequenced to date [72,73,74,75,76]. Members of *Colletotrichum* belong to six independent species complexes that deploy various infection strategies, including biotrophic, necrotrophic, hemibiotrophic, and endophytic strategies, to derive nutrients from colonized monocotyledonous and dicotyledonous plants [7,19,74]. These genomes have been intensively analyzed to identify the genomic features associated with host adaptation mechanisms. The results indicate that *Colletotrichum* species secrete specific cell wall-degrading enzymes and proteinases depending on their infection strategies. The structures of their genomes can probably be grouped based on similarities in the host range, rather than phylogenetic similarities [19,74,77]. Nevertheless, further research is needed to characterize genes associated with virulence factors in specific host plants.

This study provides comprehensive information on the structure and organization of the four newly sequenced mitochondrial genomes of *C. fioriniae*, C. *lupini, C. salicis,* and *C. tamarilloi*. The comparative study with mitogenome sequences of seven other *Colletotrichum* species revealed a high consistency in gene composition and order and also in the presence of variable regions with various possible applications. The results of this study indicate that the genes grouped within mitochondrial genomes can facilitate the development of effective methods for detecting polyphagous pathogens. In particular, the development of an effective qPCR detection method based on mitochondrial sequences appears to be a key focus for future research.

## Figures and Tables

**Table 1:** GenBank accession numbers and references for *Colletotrichum* cp genomes used in this study.

Species	Complex	Accession number	Reference
*C. acutatum*	acutatum	NC_027280	Kim et al. 2016 [27]
*C. lupini*		NC_029213	This study
*C. fioriniae*		NC_030052	This study
*C. salicis*		NC_035496	This study
*C. tamarilloi*		NC_029706	This study
*C. gloeosporioides*	gloeosporioides	KX885104	Liang et al. 2017 [28]
*C. aenigma*		KX885105	Liang et al. 2017 [28]
*C. fructicola*		KX034082	Liang et al. 2017 [28]
*C. siamense*		KX885102	Liang et al. 2017 [28]
*C. graminicola*	graminicola	NW_007361658	Vaillancourt et al. 2015 [29]
*C. lindemuthianum*	orbiculare	NC_023540	Gutierrez et al. 2016 [30]

**Table 2:** General features of the mitochondrial genomes of selected *Colletotrichum* species.

Species	Length [bp]		%GC	Start codon				Stop codon		PCGs	rRNAs	tRNAs	Introns
	Total	CDS		AUG	UUG	AUU		UAA	UAG				
*C. acutatum*	30,892	14,424	30.51	15	0	0		14	1 (*nad6*)	15	2	28	1
*C. lupini*	36,554	14,421	29.91	15	0	0		14	1 (*nad6*)	15	2	29	1
*C. fioriniae*	30,020	14,436	30.04	15	0	0		14	1 (*nad6*)	15	2	29	1
*C. salicis*	33,950	14,424	30.44	15	0	0		14	1 (*nad6*)	15	2	29	2
*C. tamarilloi*	30,824	14,421	30.50	15	0	0		14	1 (*nad6*)	15	2	29	1
*C. gloeosporioides*	55,169	14,721	34.55	14	1 (*cox3*)	0		13	2 (*cox3* and *nad6*)	15	2	27	8
*C. aenigma*	57,252	14,748	34.28	14	1 (*cox3*)	0		13	2 (*cox3* and *nad6*)	15	2	27	9
*C. fructicola*	56,051	14,475	34.04	14	1 (*cox3*)	0		13	2 (*cox3* and *nad6*)	15	2	27	9
*C. siamense*	54,645	14,745	34.30	14	1 (*cox3*)	0		13	2 (*cox3* and *nad6*)	15	2	27	7
*C. graminicola*	39,649	16,482	29.89	15	0	1 (*atp6*)		15	1 (*nad6*)	16	2	25	2
*C. lindemuthianum*	36,957	15,012	30.88	15	0	1		14	2 (*cox1* and *nad6*)	16	2	27	2

CDS: Coding sequences; PCGs: Protein Coding Genes

## Supplementary Materials

The following are available online at https://www.mdpi.com/2073-4425/11/5/552/s1, Table S1: List of the mitochondrial genomes used in the phylogenetic analysis. Species list arranged alphabetically; Table S2: Codon usage frequencies in analyzed mitogenomes of *Colletotrichum* species; Table S3a: Ks and Ka values of the *Colletotrichum acutatum* mitochondrial genome vs. selected representatives of the *Colletotrichum* genus; Table S3b: Ks and Ka values and pairwise Ka/Ks ratio for *rps3* between all *Colletotrichum* species combinations; Table S4: List of repeated sequences in the mitochondrial genomes of *Colletotrichum* sp. Genomes; Table S5a: Distribution of simple sequence repeats (SSR) in the *Colletotrichum gloeosporioides* mt genome; Table S5b: Distribution of SSR in the *Colletotrichum fructicola* mt genome; Table S5c: Distribution of SSR in the *Colletotrichum siamense* mt genome; Table S5d: Distribution of SSR in the *Colletotrichum aenigma* mt genome; Table S5e: Distribution of SSR in the *Colletotrichum acutatum* mt genome; Table S5f: Distribution of SSR in the *Colletotrichum lupini* mt genome; Table S5g: Distribution of SSR in the *Colletotrichum fioriniae* mt genome; Table S5h: Distribution of SSR in the *Colletotrichum tamarilloi* mt genome; Table S5i: Distribution of SSR in the *Colletotrichum salicis* mt genome; Table S5j: Distribution of SSR in the *Colletotrichum lindemuthianum* mt genome; Table S5k: Distribution of SSR in the *Colletotrichum graminicola* mt genome; Table S6: Identity matrix between *rps3* genes among 46 studied fungi species;. Species list arranged alphabetically; Figure S1: Sequence identity plots (mVISTA) of 11 *Colletotrichum* species using *C. acutatum* as a reference genome. The arrows indicate the annotated genes and their transcriptional directions. Genome regions are color coded as coding and non-coding regions (blue and red, respectively). The vertical scale represents the degree of identity, which ranged from 50% to 100%; Figure S2: Phylogenetic tree based on sequences of the *rps3* gene from 11 *Colletotrichum* species, 33 other Sordariomycetes representatives, and 2 *Penicillium* sp. using Bayesian posterior probabilities (PP). Bayesian PP are given at each node; Figure S3: Multiple alignment of the *rps3* gene of 11 *Colletotrichum* species. The highly variable region between positions 529 and 751 of the alignment is presented.
