# Supplementary material for: rps3 as a Candidate Mitochondrial Gene for the Molecular Identification of Species from the Colletotrichum acutatum Species Complex
Source: Genes (Basel). 2020 May 14;11(5):552. doi: 10.3390/genes11050552 (PMC7290925; doi:10.3390/genes11050552)
Supplement: Supplementary file 1 [file genes-11-00552-s001.zip › Supplementary files.v.4b/Table S1.docx]

**Table S1.** List of mitochondrial genomes used in the phylogenetic analysis. Species list arranged alphabetically.

| **Accession** | **Organism** |
| --- | --- |
| NC_029851.1 | *Acremonium fuci* |
| NC_004514.1 | *Akanthomyces muscarius* |
| NC_023117.1 | *Annulohypoxylon stygium* |
| NC_010652.2 | *Beauveria bassiana* |
| NC_030636.1 | *Beauveria caledonica* |
| NC_022708.1 | *Beauveria pseudobassiana* |
| NC_020430.1 | *Ceratocystis cacaofunesta* |
| NC_036667.1 | *Clonostachys rosea* |
| NC_027280.1 | *Colletotrichum acutatum* |
| KX885105.1 | *Colletotrichum aenigma* |
| NC_030052.1 | *Colletotrichum fioriniae* |
| KX034082.1 | *Colletotrichum fructicola* |
| KX885104.1 | *Colletotrichum gloeosporioides* |
| NW_007361658.1 | *Colletotrichum graminicola* |
| NC_023540.1 | *Colletotrichum lindemuthianum* |
| NC_029213.1 | *Colletotrichum lupini* |
| NC_035496.1 | *Colletotrichum salicis* |
| KX885102.1 | *Colletotrichum siamense* |
| NC_029706.1 | *Colletotrichum tamarilloi* |
| NC_022834.1 | *Cordyceps militaris* |
| NC_022681.1 | *Fusarium circinatum* |
| NC_036106.1 | *Fusarium commune* |
| NC_026993.1 | *Fusarium culmorum* |
| NC_025928.1 | *Fusarium gerlachii* |
| NC_009493.1 | *Fusarium graminearum* |
| NC_017930.1 | *Fusarium oxysporum* |
| NC_016680.1 | *Fusarium solani* |
| NC_016687.1 | *Fusarium verticillioides* |
| NC_027660.1 | *Hirsutella minnesotensis* |
| NC_030164.1 | *Hirsutella rhossiliensis* |
| NC_036610.1 | *Hirsutella vermicola* |
| NC_030340.1 | *Ilyonectria destructans* |
| NC_028330.1 | *Lecanicillium saksenae* |
| NC_008068.1 | *Metarhizium anisopliae* |
| NC_030252.1 | *Nectria cinnabarina* |
| NC_034659.1 | *Ophiocordyceps sinensis* |
| NC_032302.1 | *Parengyodontium album* |
| NC_030172.1 | *Penicillium polonicum* |
| NC_027416.1 | *Penicillium roqueforti* |
| NC_031828.1 | *Pestalotiopsis fici* |
| NC_022835.1 | *Pochonia chlamydosporia* |
| NC_036382.1 | *Tolypocladium inflatum* |
| NC_031384.1 | *Tolypocladium ophioglossoides* |
| NC_036144.1 | *Trichoderma hamatum* |
| NC_008248.1 | *Verticillium dahliae* |
| NC_029238.1 | *Verticillium nonalfalfae* |
