# Supplementary figures and images for: rps3 as a Candidate Mitochondrial Gene for the Molecular Identification of Species from the Colletotrichum acutatum Species Complex
Source: Genes (Basel). 2020 May 14;11(5):552. doi: 10.3390/genes11050552 (PMC7290925; doi:10.3390/genes11050552)

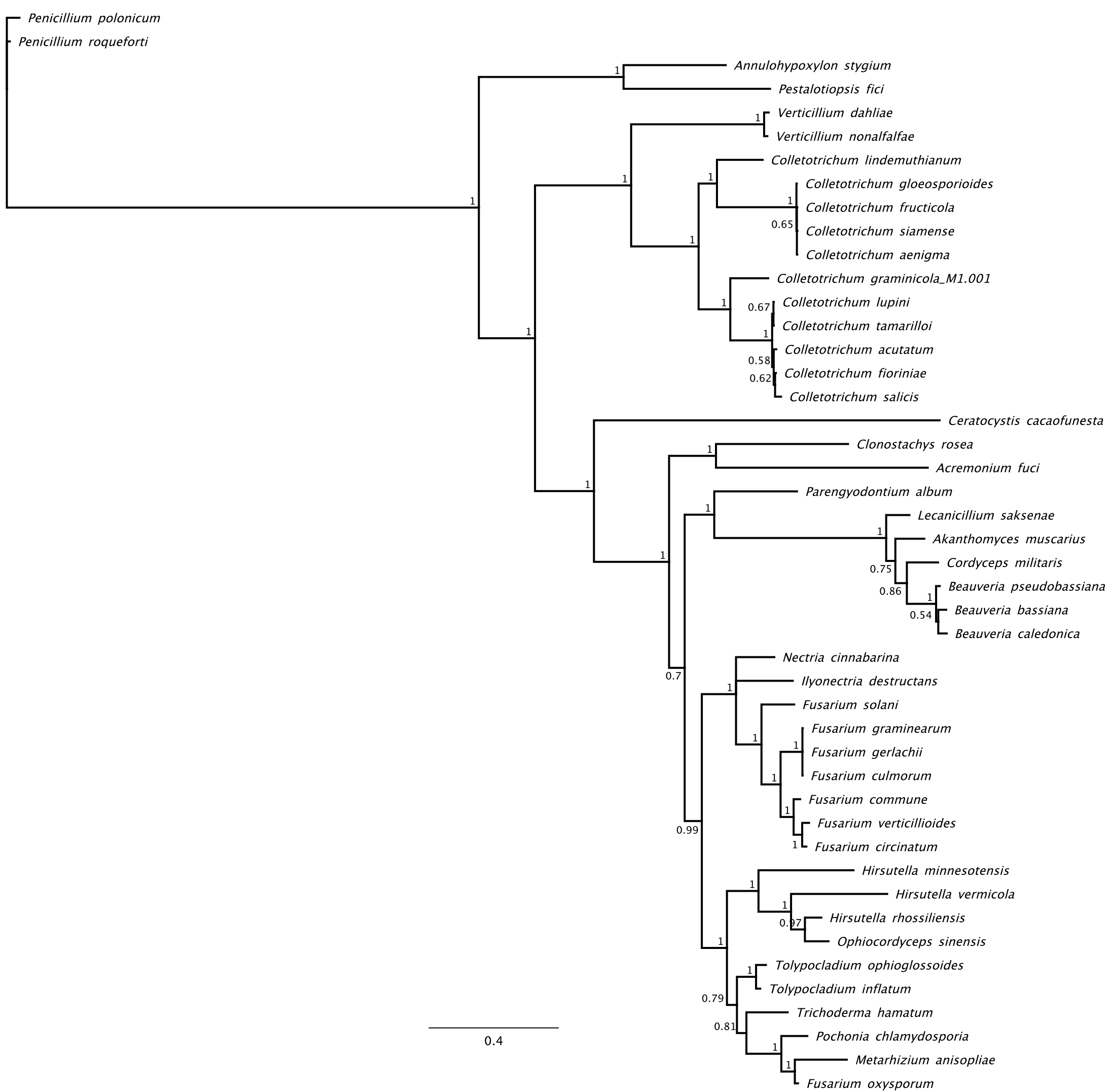

Supplement: Supplementary file 1 [file genes-11-00552-s001.zip › Supplementary files.v.4b/Figure_S2.pdf]

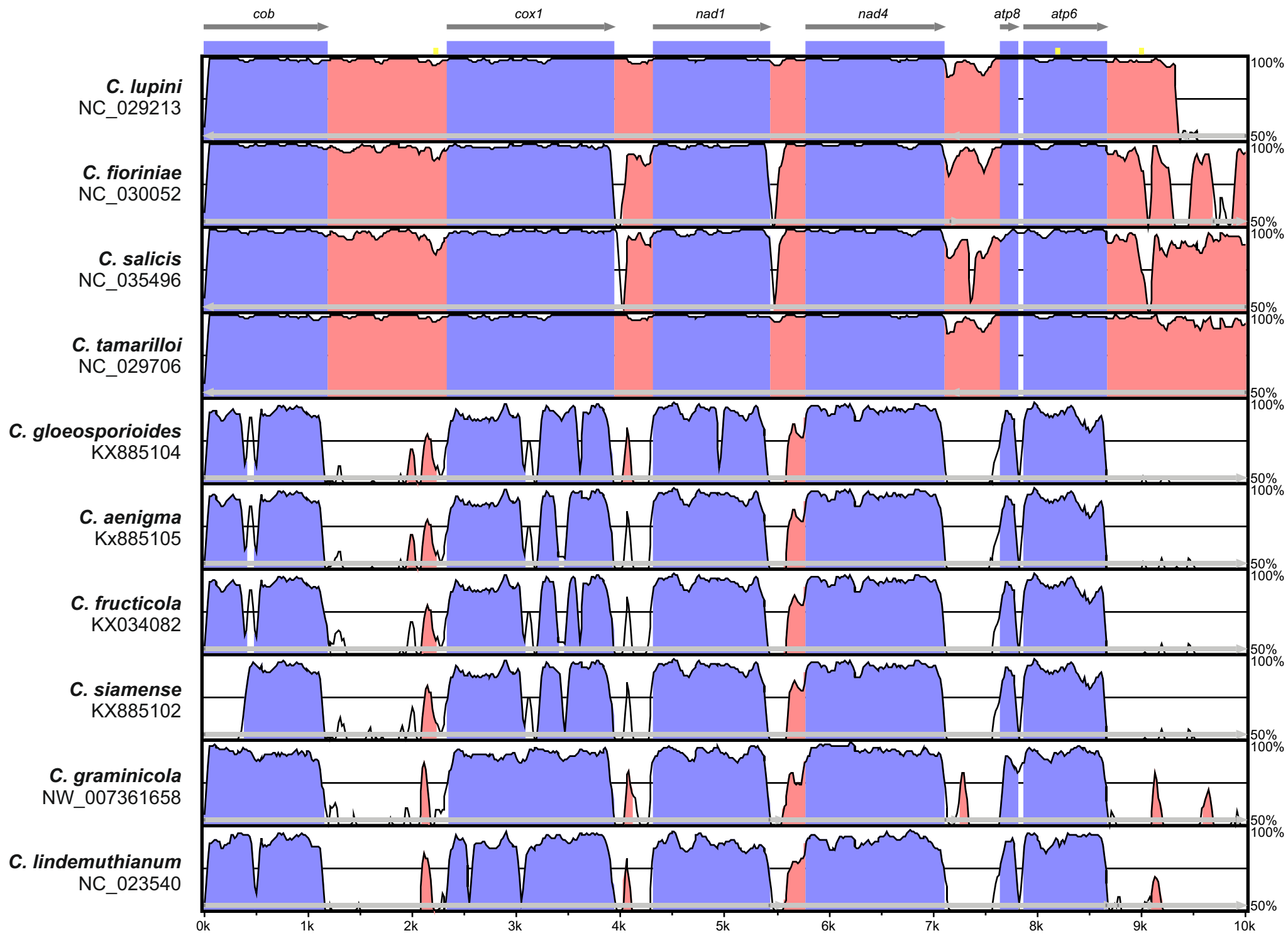

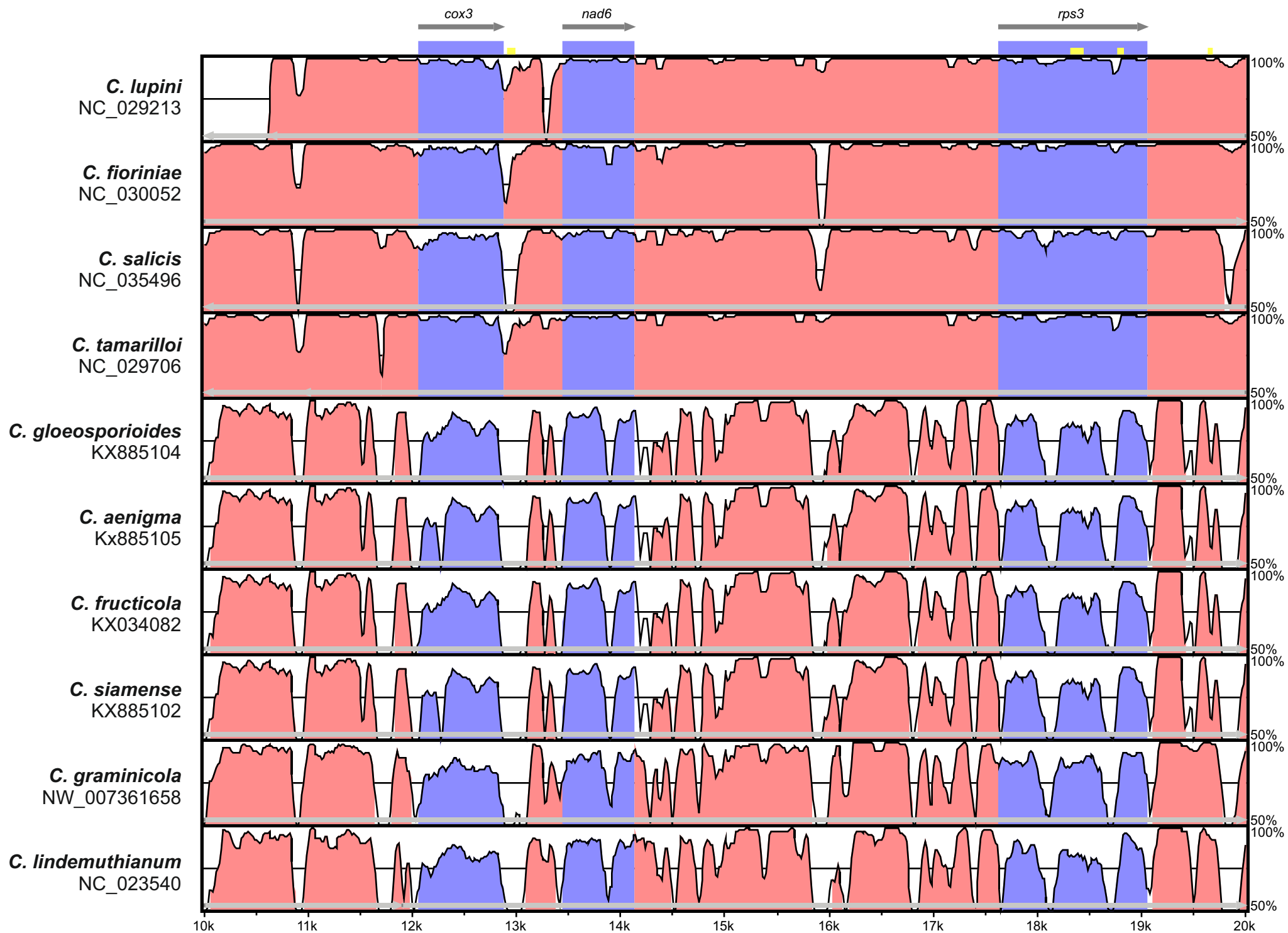

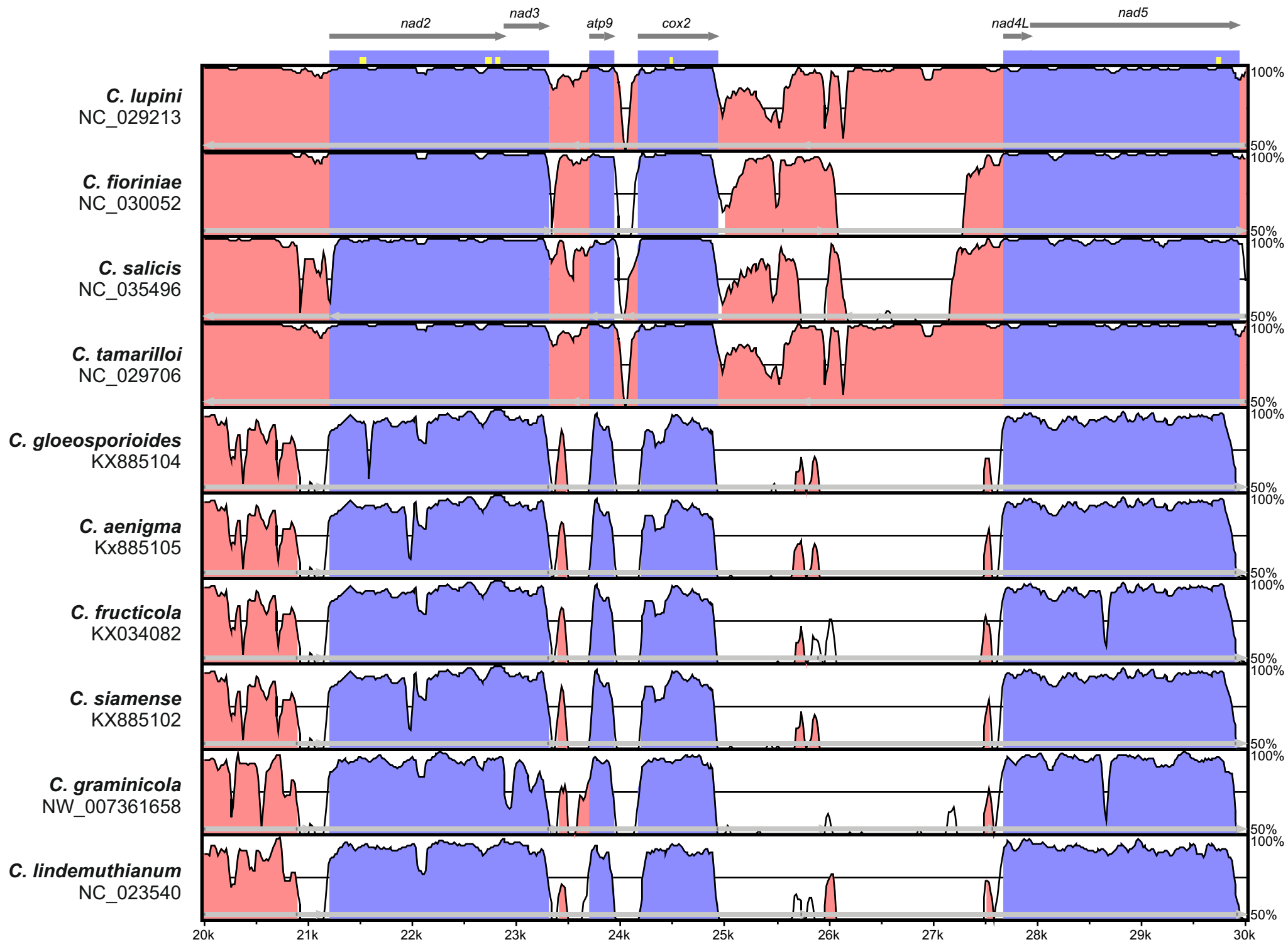

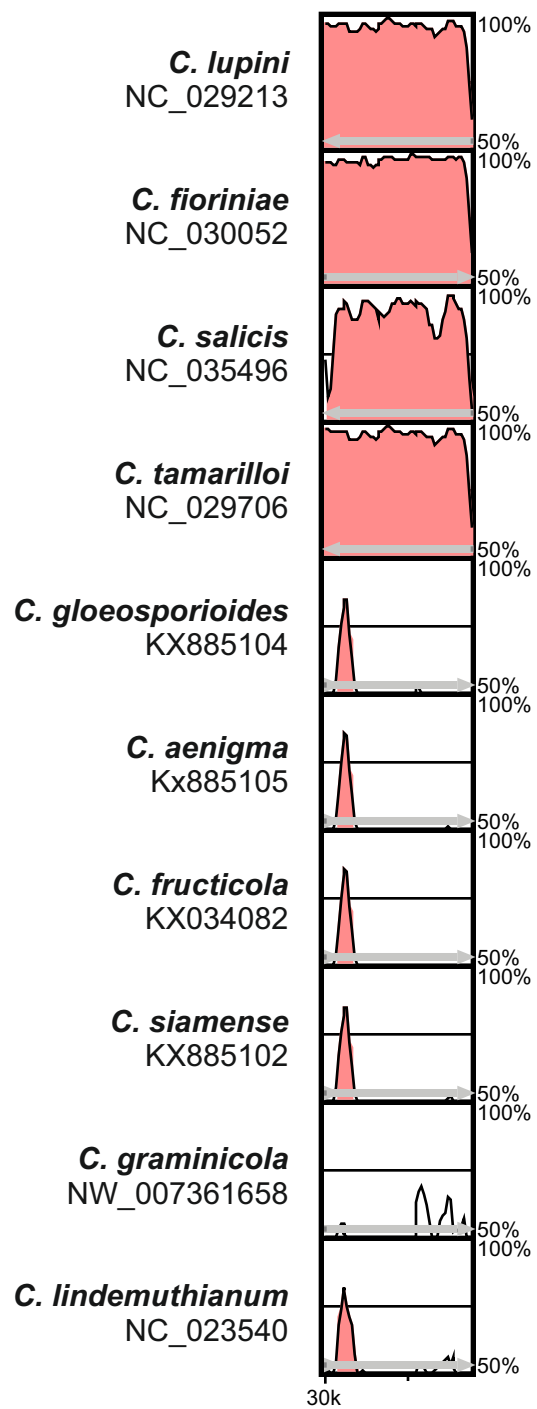

Supplement: Supplementary file 1 [file genes-11-00552-s001.zip › Supplementary files.v.4b/Figure_S1.pdf]
