# Supplementary material for: rps3 as a Candidate Mitochondrial Gene for the Molecular Identification of Species from the Colletotrichum acutatum Species Complex
Source: Genes (Basel). 2020 May 14;11(5):552. doi: 10.3390/genes11050552 (PMC7290925; doi:10.3390/genes11050552)
Supplement: Supplementary file 1 [file Figure_S3.pdf › Supplementary files.v.4b/Figure_S3.pdf]

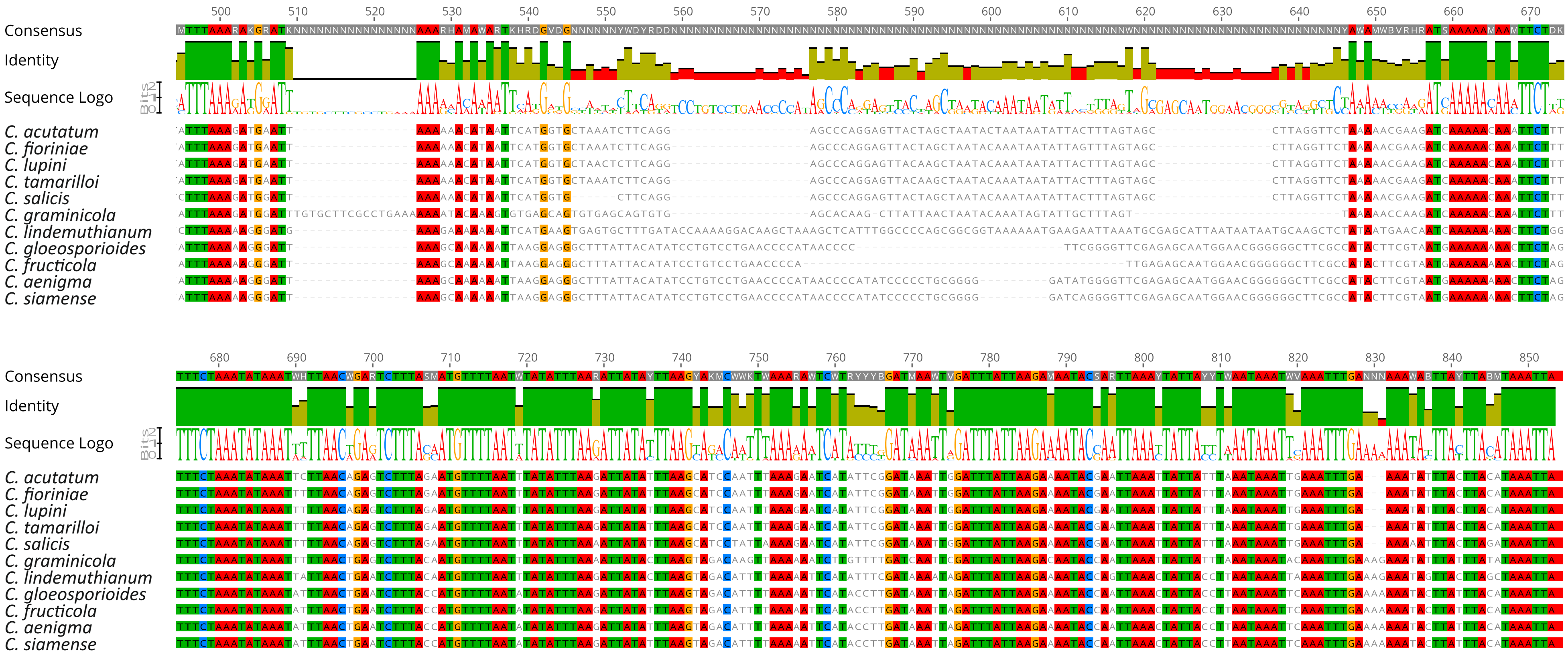

500
510
520
530
540
550
560
570
580
590
600
610
620
630
640
650
660
670
Consensus
Identity
Sequence Logo
Bits
C. acutatum
C. fioriniae
C. lupini
C. tamarilloi
C. salicis
C. graminicola
C. lindemuthianum
C. gloeosporioides
C. fructicola
C. aenigma
C. siamense
680
690
700
710
720
730
740
750
760
770
780
790
800
810
820
830
840
850
Consensus
Identity
Sequence Logo
Bits
C. acutatum
C. fioriniae
C. lupini
C. tamarilloi
C. salicis
C. graminicola
C. lindemuthianum
C. gloeosporioides
C. fructicola
C. aenigma
C. siamense
